# Hepatitis B relapse after entecavir or tenofovir alafenamide cessation under anti-viral prophylaxis for cancer chemotherapy

**DOI:** 10.1186/s12985-024-02338-6

**Published:** 2024-04-03

**Authors:** Hsin-Wei Fang, Po-Lin Tseng, Tsung-Hui Hu, Jing-Houng Wang, Chao-Hung Hung, Sheng-Nan Lu, Chien-Hung Chen

**Affiliations:** Division of Hepatogastroenterology, Department of Internal Medicine, Kaohsiung Chang Gung Memorial Hospital, Chang Gung University College of Medicine, 123 Ta Pei Road, Kaohsiung, Taiwan

**Keywords:** Entecavir, Tenofovir alafenamide, Rituximab, Chemotherapy, Chronic hepatitis B

## Abstract

**Background:**

No study has comparing hepatitis B virus (HBV) relapse rates among patients with both cancer and hepatitis B e antigen (HBeAg)-negative chronic hepatitis B (CHB) who completed anti-viral prophylaxis for chemotherapy and then stopped taking entecavir or tenofovir alafenamide (TAF).

**Methods:**

A total of 227 HBeAg-negative cancer patients without cirrhosis who previously took entecavir (*n* = 144) or TAF (*n* = 83) for antiviral prophylaxis were enrolled.

**Results:**

The cumulative incidence of virological and clinical relapse at 2 years was 37% and 10.4%, respectively, in the entecavir group, and 46.7% and 19.5%, respectively, in the TAF group. The multivariate analysis revealed that the use of hematologic malignancy, TAF use, and high-viremia group at baseline were independent risk factors for virological relapse, and use of rituximab, TAF use, higher FIB-4 index and high-viremia group at baseline were independent risk factors for clinical relapse. After propensity score-matching, the patients who discontinued TAF therapy still exhibited higher virological (*P* = 0.031) and clinical relapse rates (*P* = 0.012) than did those who discontinued entecavir therapy. The patients were allocated to high- (> 2000 IU/mL), moderate- (between 20 and 2000 IU/mL) and low- (< 20 IU/mL) viremia groups. In the high-viremia group, those who had taken TAF for antiviral prophylaxis had higher rates of virological and clinical relapse than did those who had taken entecavir; in the moderate- and low-viremia groups, no significant difference in virological and clinical relapse rates was detected between the entecavir and TAF groups. Three patients experienced hepatic decompensation upon clinical relapse. All three patients were lymphoma and underwent rituximab therapy. One patient developed acute on chronic liver failure and died even though timely retreatment.

**Conclusions:**

In patients with both cancer and CHB who underwent antiviral prophylaxis, TAF use was associated with a higher chance of HBV relapse than entecavir use after nucleos(t)ide analogue cessation, particularly in the high-viremia group. Patients who are hematologic malignancy and undergo a rituximab-containing cytotoxic therapy should be monitored closely after withdrawal from prophylactic NA treatment.

## Background

Because the covalently closed circular DNA of HBV serves as a template for viral replication, patients with both cancer and HBV are at risk of HBV reactivation (HBVr) when they undergo cytotoxic or immunosuppressive therapy, and this risk is notably higher in those with lymphoma [1–4]. HBVr not only causes liver-related diseases, hepatic decompensation, and mortality but also disrupts anticancer therapy [4]. Accordingly, clinical guidelines have recommended prophylactic nucleos(t)ide analogue (NA) therapy for patients with both cancer and chronic hepatitis B (CHB) before they undergo anticancer management [5–8].

Of the numerous NA therapies available, entecavir and tenofovir disoproxil fumarate (TDF) have demonstrated comparable effectiveness in suppressing HBV and reducing HBVr in patients with CHB who have undergone anticancer treatment for oncologic and hematologic diseases on the basis of a high genetic barrier [9, 10]. Tenofovir alafenamide (TAF), a novel prodrug of tenofovir, provides greater stability than does TDF, thereby enabling HBV suppression at a lower dose and with less renal toxicity and mineral bone density loss [11–13]. Studies have reported that TAF exhibited comparable antiviral efficacy to entecavir and TDF in patients undergoing chemotherapy or switching from TDF during anticancer treatments [14, 15].

Prophylactic NA therapy can reduce the risk of HBVr during anticancer therapy; however, in a 1-year follow-up conduced after the cessation of antiviral prophylaxis, the rate of HBV relapse was approximately 14.3% and 10.8% for TDF and entecavir, respectively [10]. Several studies have also demonstrated that in patients with CHB, TDF therapy is associated with a higher rate and earlier occurrence of HBV relapse than entecavir therapy after NA withdrawal [16–18]. However, no study has reported on HBV relapse rates after the cessation of TAF as an antiviral prophylaxis. This study compared the HBV relapse rates of patients with cancer and hepatitis B e antigen (HBeAg)-negative CHB after prophylactic entecavir or TAF therapy for chemotherapy.

## Patients and methods

### Patients

This retrospective study enrolled 144 HBeAg-negative patients without cirrhosis who underwent entecavir treatment between 2012 and 2017 and 83 HBeAg-negative patients without cirrhosis who underwent TAF treatment between 2019 and 2022; for these patients the entecavir or TAF were administrated for antiviral prophylaxis before anticancer management for oncologic and hematologic malignancies, and clinical posttreatment follow-up was conducted for > 6 months.

Each diagnosis of malignancy was confirmed through histological and imaging tests and by an oncology specialist. The chemotherapy regimens for each patient were determined by clinical oncologic and hematologic specialists in accordance with a standard protocol.

All the patients had been HBsAg positive for > 6 months and had undergone prophylactic entecavir or TAF therapy within 1 week of chemotherapy initiation. In accordance with the criteria established by Taiwan’s National Health Insurance system, the patients completed additional NA therapies for 6 months after chemotherapy (including anthracycline or rituximab-containing agents). All patients exhibited undetectable HBV DNA levels (< 20 IU/mL) at the end of treatment.

Patients were excluded if they had autoimmune hepatitis, alcoholic liver disease, or coinfection with hepatitis C virus (HCV), hepatitis D virus, or human immunodeficiency virus. In addition, patients who lost HBsAg or had HCC or cirrhosis during treatment were excluded.

Non-cirrhosis was diagnosed on the basis of combined repeated ultrasound findings that indicated the absence of clinical features such as splenomegaly, gastroesophageal varices, or ascites at baseline. The present study was conducted in accordance with the Declaration of Helsinki of 1975 and approved by the Research Ethics Committees of Chang Gung Memorial Hospital (Kaohsiung, Taiwan). Informed consent was obtained from all participants.

### Methods

The patients were followed up every 1–3 months while they underwent antiviral prophylaxis with entecavir or TAF therapy. The patients’ serum HBV DNA was assayed every 3–6 months during the antiviral treatment.

After either entecavir or TAF therapy, the patients were monitored every 1–3 months during the first 6 months and then every 3 months until retreatment or their final hospital visit. Additional biweekly or weekly visits were arranged if their alanine aminotransferase (ALT) levels increased beyond the upper limit of normal (ULN). The patients’ serum HBV DNA was monitored every 1–3 months for the first 6 months after cessation of therapy and then every 3–6 months thereafter. Additional HBV DNA tests were conducted when virological or clinical relapse occurred. The monitoring interval after cessation of entecavir or TAF therapy was similar between entecavir and TAF groups.

### Definition of HBV relapse after NA withdrawal

A virological relapse was defined as the reappearance of a serum HBV DNA level of ≥ 2000 IU/mL after the cessation of NA therapy. A clinical relapse was defined as an episode of ALT elevation (≥ 2 × ULN) accompanied by an HBV DNA of ≥ 2000 IU/mL after the cessation of NA therapy [19]. The consolidation duration was calculated from the cessation of chemotherapy to the end of NA treatment. The FIB-4 index was calculated using the following formula: aspartate—aminotransferase (AST) [IU/L] × age [years]/platelet count [10^9^/L] × alanine aminotransferase (ALT) [IU/L]^1/2^ [20].

### Serology

The presence of HBsAg, HBeAg, and anti-HCV antibodies was determined using commercial assay kits (HBsAg EIA, Abbott, North Chicago, IL, USA; HBeAg EIA, Abbott; anti-HCV, EIA 3.0, Abbott). Serum HBV DNA was quantified using the COBAS TaqMan HBV test (CAP-CTM; Roche Molecular Systems, Branchburg, NJ, USA), with the lower limit of detection being 20 IU/Ml.

### Statistical analysis

Demographic and clinical data are presented as means ± standard deviations and medians (range) for normally and non-normally distributed continuous variables, respectively, whereas categorical variables are presented as number of cases (proportional values). To compare the groups, chi-square tests were performed to analyze categorical variables, and Student’s *t*-tests and Mann–Whitney U tests were performed to analyze normal and non-normal continuous variables, respectively. Kaplan–Meier analysis with log-rank testing was conducted to compare the cumulative incidences of posttreatment virological and clinical relapse between the entecavir and TAF groups. Cox proportional hazards regression models were applied using the forward method to identify independent factors associated with post-treatment virological and clinical relapse; the variables that appeared to be significant in a univariate analysis (< 0.05) or clinically significant were subjected to multivariate analysis for adjustment. All statistical tests were 2 sided, and significance was indicated by a *P* value of 0.05.

Propensity score matching was conducted to reduce the significant differences in clinical features between the off-TAF and off-entecavir groups at a 1:1 to 1:2 ratio, and adjustments were made for age, sex, hematologic or solid malignancy, use of rituximab, steroid or anthracycline use, baseline ALT and HBV DNA levels, and treatment and consolidation duration. Caliper matching was conducted for propensity scores (nearest available matching). Patients’ pairs (discontinuing and continuing groups) were propensity score matched such that the difference with the logit of the propensity scores of the 2 groups would be within a standard deviation range of 0.2 [21, 22]. All statistical analyses were performed using SPSS 25.0.

## Results

### Clinical characteristics of study population

The 227 patients had a median antiviral prophylaxis duration of 52 weeks. The clinical characteristics of the entecavir and TAF groups are presented in Table 1. Of the 27 patients with hematologic malignancy, 23 were lymphoma and 20 received rituximab therapy. There was no significant difference in clinical features between entecavir and TAF groups.

**Table 1:** Clinical characteristics of study population

Variables, n (%) or mean ± S.D (range)	Entecavir N = 144	TAF N = 83	*p* value
Age (years)	54.5 ± 10.5	55.5 ± 9.8	0.473
Gender (male: female), n	44:100	34:49	0.112
Use of rituximab, n (%)	16 (11.1%)	6 (7.2%)	0.341
Use of steroid, n (%)	15 (10.4%)	4 (4.8%)	0.142
Use of anthracycline, n (%)	51 (35.4%)	25 (30.1%)	0.415
Hematologic malignancy, n (%)	17 (11.8%)	10 (12%)	0.957
Distant metastasis	21 (14.6%)	16 (19.2%)	0.356
Liver metastasis	7 (4.9%)	3 (3.6%)	0.750
AST (U/L)	35.0 ± 55.8	28.4 ± 19.5	0.298
ALT (U/L)	43.0 ± 96.8	31.6 ± 32.3	0.299
Total bilirubin (mg/dL)	0.63 ± 0.38	0.71 ± 0.46	0.153
Platelet × 10^3^/μL	248.4 ± 83.8	250.3 ± 102.7	0.881
FIB-4	1.39 ± 0.94	1.42 ± 0.91	0.822
HBV DNA (log_10_ IU/mL)	2.85 ± 1.66	2.80 ± 1.54	0.836
Low-viremia group, n (%)	36 (25%)	24 (28.9%)	0.541
Moderate-viremia group, n (%)	61 (42.4%)	29 (34.9%)	
High-viremia group, n (%)	47 (32.6%)	30 (36.1%)	
Treatment duration (weeks)	60.6 ± 25.1	55.7 ± 18.6	0.116
Consolidation duration (weeks)	30.2 ± 10.8	31.1 ± 10.8	0.555

*ALT* Alanine transaminase, *AST* Aspartate transaminase, *SD* standard deviation, *TAF* tenofovir alafenamide

High-viremia group: Baseline HBV DNA levels of > 2000 IU/mL, Moderate-viremia group: baseline HBV DNA levels of ≧20 and < 2000 IU/mL, Low-viremia group: baseline HBV DNA levels of < 20 IU/mL

### Incidence and predictors of virological relapse and clinical relapse after NA cessation

**Fig. 1:**
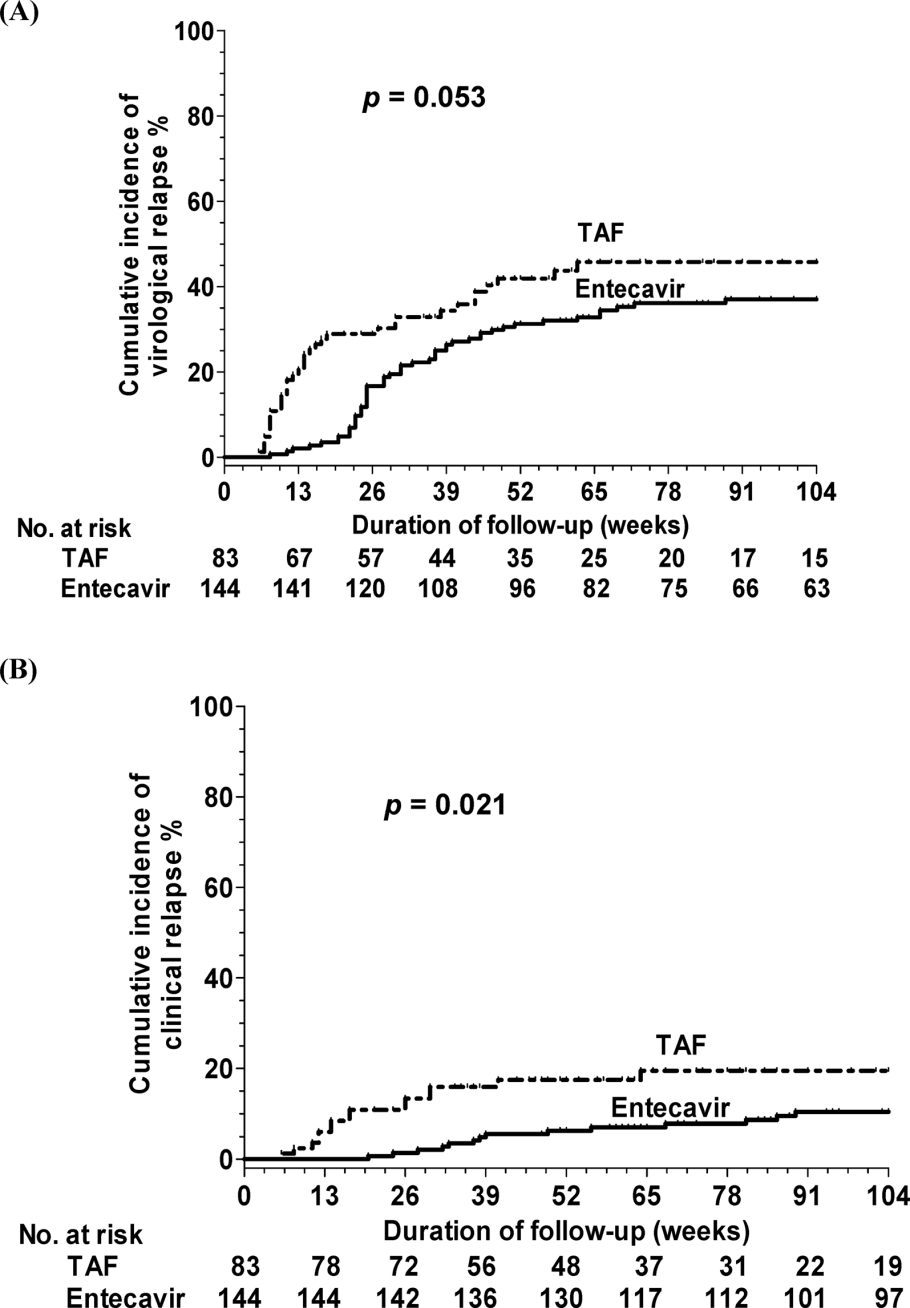
Cumulative incidences of HBV relapse after cessation of antiviral prophylactic entecavir and tenofovir alafenamide (TAF) therapy for all patients. **A** Virological relapse **B** clinical relapse

During the antiviral prophylaxis conducted with NA therapy, none of the patients experienced HBVr. Of the 227 patients with CHB, 57 in the entecavir group and 35 in the TAF group experienced virological relapse after NA cessation. The cumulative incidences of virological relapse at 13, 26, 52, and 104 weeks were 2.1%, 16.7%, 31.3%, and 37%, respectively, in the entecavir group, and 20.5%, 28.9%, 41.9%, and 46.7%, respectively, in the TAF group. There was borderline significant difference in virological relapse rate was detected between the entecavir and TAF groups (*P* = 0.053, Fig. 1A).

Of the 227 patients, 16 in the entecavir group and 15 in the TAF group experienced clinical relapse after NA cessation. The cumulative incidences of clinical relapse at 13, 26, 52, and 104 weeks were 0%, 1.4%, 6.3%, and 10.4%, respectively, in the entecavir group, and 6%, 13.3%, 17.4%, and 19.5%, respectively, in the TAF group. There was a significant difference in clinical relapse between the entecavir and TDF groups (*P* = 0.021, Fig. 1B).

The univariate and multivariate analysis results for virological and clinical relapse are presented in Tables 2 and 3, respectively. The multivariate analysis revealed that the use of hematologic malignancy, TAF use, and high-viremia group at baseline were independent risk factors for virological relapse, and use of rituximab, TAF use, higher FIB-4 index and high-viremia group at baseline were independent risk factors for clinical relapse.

**Table 2:** Factors predictive of virological relapse after entecavir or TAF cessation for all patients

Variables	Comparison	Univariate analysis		Multivariate analysis	
		HR (95% CI)	*p* value	HR (95% CI)	*p* value
Age (years)	Increase per one year	0.991 (0.972–1.011)	0.383		
Gender	Male vs. female	0.9523 (0.603–1.414)	0.713		
Use of Rituximab	Yes vs. no	2.123 (1.201–3.752)	0.010		
Use of steroid	Yes vs. no	1.964 (1.070–3.606)	0.029		
Use of anthracycline	Yes vs. no	1.346 (0.885–2.047)	0.164		
Hematologic malignancy	Yes vs. no	2.326 (1.372–3.943)	0.002	2.034 (1.178–2.513)	0.011
Distant metastasis	Yes vs. no	1.032 (0.594–1.795)	0.910		
NA therapy	TAF vs. entecavir	1.231 (0.994–1.525)	0.056	1.386 (1.109–1.732)	0.004
AST (U/L)	Increase per one U/L	1.003 (1.001–1.006)	0.019		
ALT (U/L)	Increase per one U/L	1.002 (1.001–1.004)	0.008		
Total bilirubin	Increase per one mg/dL	1.251 (0.749–2.087)	0.392		
Platelet × 10^3^/μL	Increase per 10^3^/μL	0.998 (0.996–1.001)	0.146		
FIB-4	Increase per ratio	1.192 (0.989–1.436)	0.065		
Viremia groups*			< 0.001		< 0.001
Low-viremia group		Reference		Reference	
Moderate-viremia group		1.670 (0.802–3.479)	0.171	1.671 (0.802–3.482)	0.171
High-viremia group		6.983 (3.553–13.724)	< 0.001	6.975 (3.533–13.769)	< 0.001
HBsAg level at EOT (IU/mL)**	Increase per one log IU/mL	2.285 (1.825–2.860)	< 0.001		
Treatment duration	Increase per one week	1.001 (0.992–1.011)	0.759		
Consolidation duration	Increase per one week	1.009 (0.991–1.027)	0.352		

*ALT* Alanine transaminase, *CI* confidence interval, *HR* hazard ratio, *NA* nucleoside analogues, *TAF* tenofovir alafenamide

*High-viremia group: Baseline HBV DNA levels of > 2000 IU/mL, Moderate-viremia group: baseline HBV DNA levels of ≧20 and < 2000 IU/mL, Low-viremia group: baseline HBV DNA levels of < 20 IU/mL

**Only 207 patients had serum available for HBsAg measurement at EOT. Multivariable analysis did not include HBsAg level at EOT due to missing data

**Table 3:** Factors predictive of clinical relapse after entecavir or TAF cessation for all patients

Variables	Comparison	Univariate analysis		Multivariate analysis	
		HR (95% CI)	*p* value	HR (95% CI)	*p* value
Age (years)	Increase per one year	1.019 (0.985–1.054)	0.279		
Gender	Male vs. female	0.486 (0.240–0.983)	0.045		
Use of Rituximab	Yes vs. no	4.469 (2.057–9.710)	< 0.001	9.831 (4.007–24.121)	< 0.001
Use of steroid	Yes vs. no	2.903 (1.190–7.079)	0.019		
Use of anthracycline	Yes vs. no	0.779 (0.359–1.691)	0.527		
Hematologic malignancy	Yes vs. no	4.525 (2.128–9.639)	< 0.001		
Distant metastasis	Yes vs. no	1.368 (0.561–3.334)	0.491		
NA therapy	TAF vs. entecavir	1.505 (1.053–2.150)	0.025	2.114 (1.415–3.158)	< 0.001
AST (U/L)	Increase per one U/L	1.007 (1.004–1.010)	< 0.001		
ALT (U/L)	Increase per one U/L	1.004 (1.003–1.006)	< 0.001		
Total bilirubin	Increase per one mg/dL	1.668 (0.788–3.527)	0.181		
Platelet × 10^3^/μL	Increase per 10^3^/μL	0.993 (0.988–3.527)	0.014		
FIB-4	Increase per ratio	1.770 (1.429–2.193)	< 0.001	1.970 (1.557–2.492)	< 0.001
Viremia groups*			< 0.001		< 0.001
Low-viremia group		Reference		Reference	
Moderate-viremia group		1.230 (0.307–4.920)	0.770	1.299 (0.318–5.299)	0.715
High-viremia group		6.091 (1.823–20.352)	0.003	7.259 (2.075–25.401)	0.002
HBsAg level at EOT (IU/mL)**	Increase per one log IU/mL	2.050 (1.384–3.038)	< 0.001		
Treatment duration	Increase per one week	1.002 (0.988–1.017)	0.771		
Consolidation duration	Increase per one week	0.977 (0.941–1.013)	0.221		

*ALT* Alanine transaminase, *CI* confidence interval, *EOT* end of treatment, *HR* Hazard ratio, *NA* nucleoside analogues, *TAF* tenofovir alafenamide

*High-viremia group: baseline HBV DNA levels of > 2000 IU/mL, Moderate-viremia group: baseline HBV DNA levels of ≧ 20 and < 2000 IU/mL, Low-viremia group: baseline HBV DNA levels of < 20 IU/mL

**Only 207 patients had serum available for HBsAg measurement at EOT. Multivariable analysis did not include HBsAg level at EOT due to missing data

### Comparison of virological and clinical relapse rates between entecavir and TAF groups based on baseline HBV viral load

Because HBV DNA level at baseline was a significant factor for virological or clinical relapse, the patients were divided into 3 subgroups on the basis of their baseline HBV DNA levels; Groups I (high-viremia group), II (moderate-viremia group), and III (low-viremia group) comprised the patients with HBV DNA levels of > 2000 IU/mL, ≧ 20 and < 2000 IU/mL, and < 20 IU/mL, respectively.

**Fig. 2:**
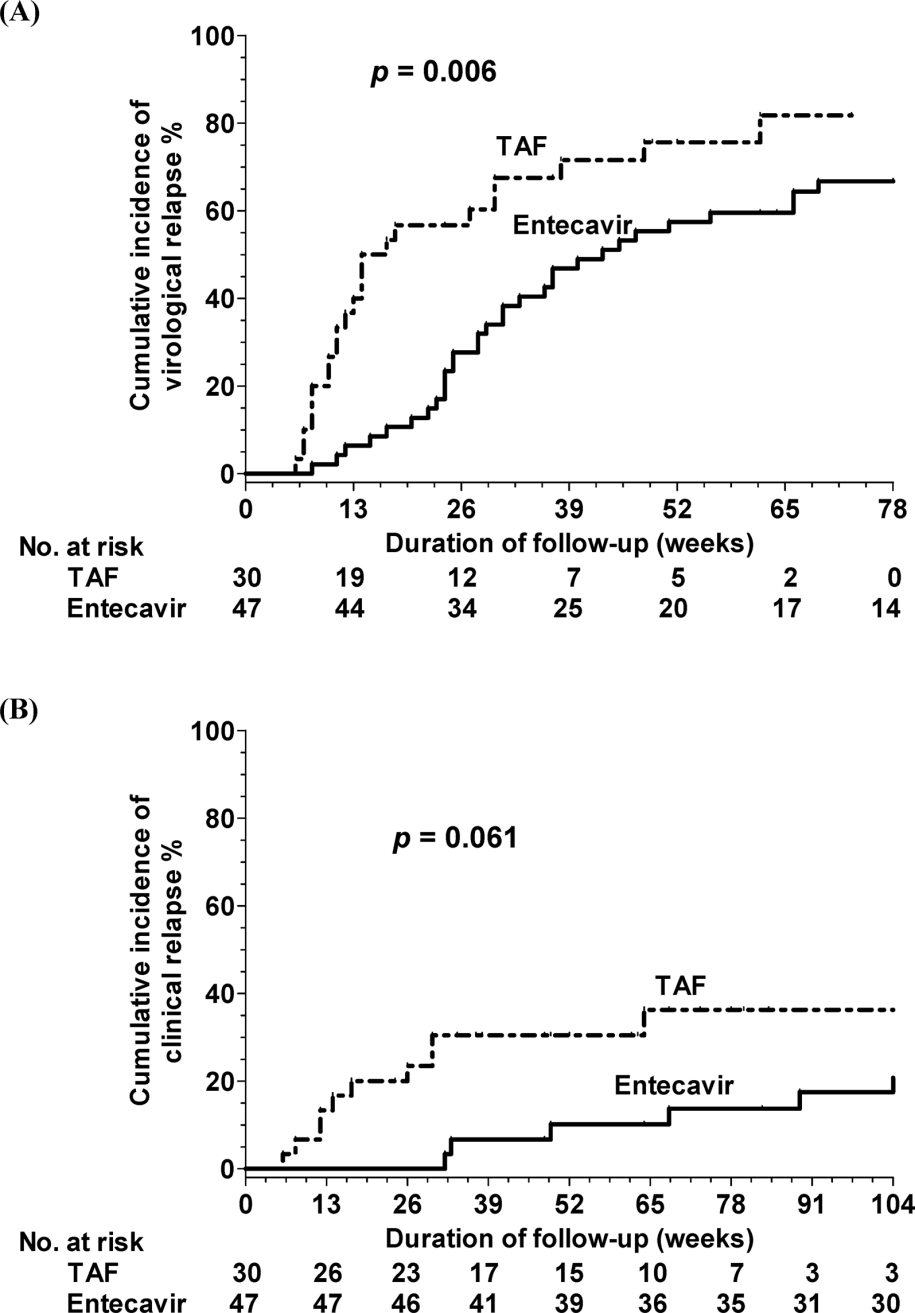
Cumulative incidences of HBV relapse after cessation of antiviral prophylactic entecavir and TAF therapy in high-viremia group (HBV DNA > 2000 IU/mL) at initial treatment (**A**) virological and (**B**) clinical relapse

In the Group I, the cumulative incidences of virological relapse at 26 and 52 weeks were 27.7% and 57.4%, respectively, for the entecavir group, and 56.7% and 75.6%, respectively, for the TAF group. A significant difference was detected in the virological relapse rates between the entecavir and TAF groups in Group I (*P* = 0.006, Fig. 2A). The cumulative incidences of clinical relapse at 26 and 52 weeks were 2.1% and 12.9%, respectively, for the entecavir group, and 23.5% and 30.4%, respectively, for the TAF group. A borderline significant difference was detected in the clinical relapse rates between the entecavir and TAF groups (*P* = 0.061, Fig. 2B). The multivariate analysis revealed that being in the TAF group was an independent factor for virological relapse (Hazard ratio [HR] 1.450; 95% confidence interval [CI]. 1.103–1.906; *P* = 0.008) and clinical relapse (HR, 2.095; 95% CI 1.264–3.471; *P* = 0.004) after adjustment for other factors.

In the Group II, the cumulative incidences of virologic relapse at 26, 52 and 104 weeks were 14.8%, 21.3% and 23.3%, respectively, for the entecavir group, and 13.8%, 28.2% and 33.0%, respectively, for the TAF group; the clinical relapse rates at 26, 52 and 104 weeks were 1.6%, 1.6% and 3.7%, respectively, for the entecavir group, and 10.3%, 13.9% and 13.9%, respectively, for the TAF group. In the Group II, no significant difference in virological relapse rate was detected between the entecavir and TAF groups (*P* = 0.383). However, there was a significant difference in clinical relapse rate between the two groups (*P* = 0.044). The multivariate analysis revealed that being in the TAF group was an independent factor for clinical relapse (HR, 4.972; 95% CI 1.399–17.673; *P* = 0.013) after adjustment for other factors.

In the Group III, the cumulative incidences of virological relapse at 26, 52 and 104 weeks were 5.6%, 13.9% and 17.1%, respectively, in the entecavir group, and 12.5%, 12.5% and 12.5%, respectively, in the TAF group. For clinical relapse, the cumulative incidences at 26, 52 and 104 weeks were 0%, 5.6% and 5.6%, respectively, in the entecavir group, and 4.2%, 4.2% and 4.2%, respectively, in the TAF group. No significance difference between the entecavir and TAF groups was detected with respect to their virological and clinical relapse rates (*P* = 0.964 and 0.966 for virological and clinical relapse rates, respectively).

### Comparisons of virological and clinical relapse rates of entecavir and TAF groups after propensity score matching

**Fig. 3:**
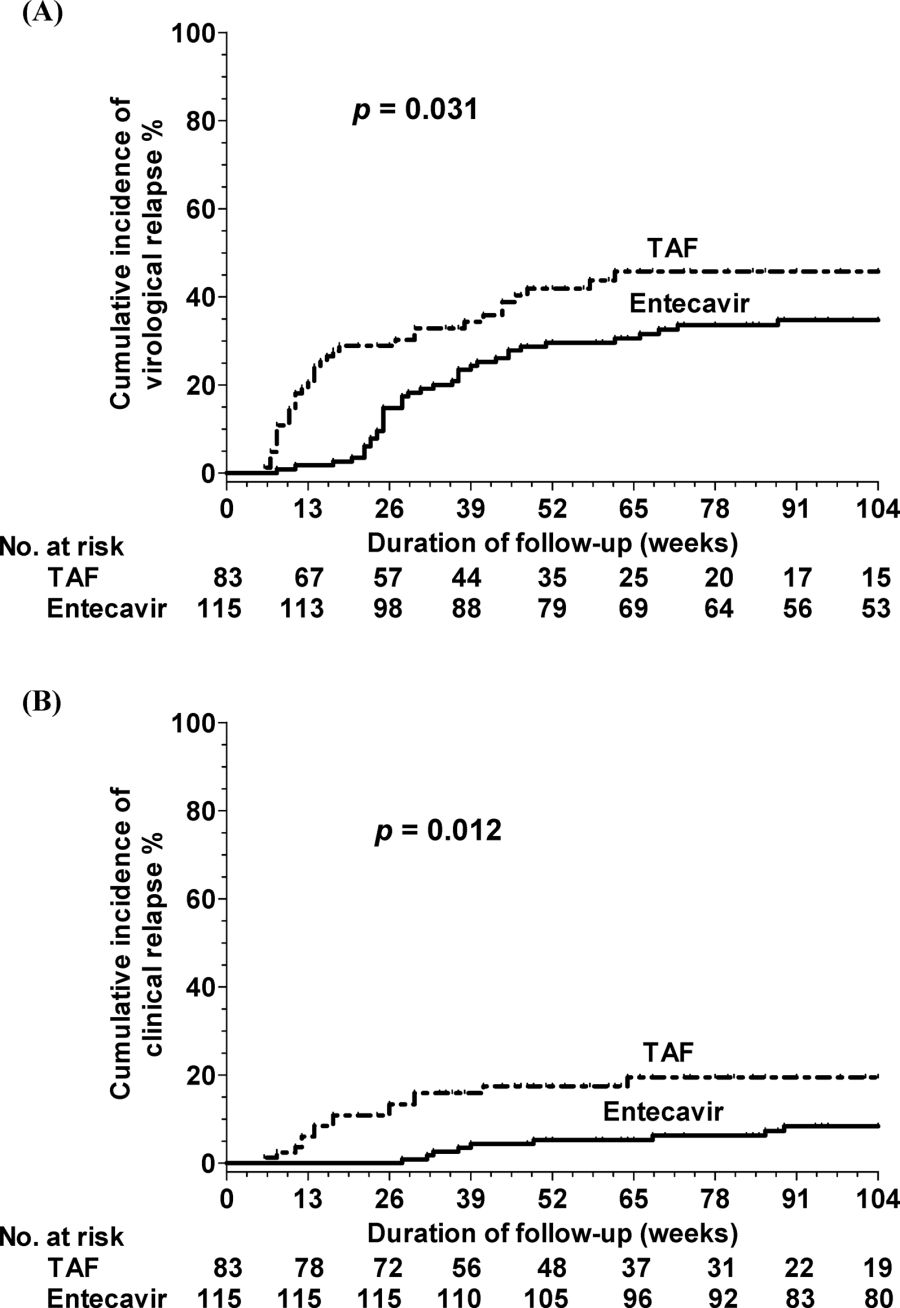
Cumulative incidences of HBV relapse after cessation of antiviral prophylactic entecavir and TAF Groups after propensity score matching for all patients. **A** virological and **B** clinical relapse

Propensity score matching yielded 115 and 83 matched patients in the entecavir and TAF groups, respectively. No significant differences in clinical features were detected between the groups (Table 4). The patients who discontinued TAF therapy exhibited significantly higher virological (*P* = 0.031) and clinical (*P* = 0.012) relapse rates than did those who discontinued entecavir therapy (Fig. 3).

**Table 4:** Clinical characteristics of study population after propensity score matching for all patients

Variables, n (%) or mean ± S.D (range)	Entecavir N = 115	TAF N = 83	*p* value
Age (years)	55.1 ± 10.8	55.5 ± 9.8	0.783
Gender (male: female), n	38:77	34:49	0.253
Use of rituximab, n (%)	11 (9.6%)	6 (7.2%)	0.563
Use of steroid, n (%)	11 (9.6%)	4 (4.8%)	0.213
Use of anthracycline, n (%)	40 (34.8%)	25 (30.1%)	0.491
Hematologic malignancy, n (%)	12 (10.4%)	10 (12%)	0.722
Distal metastasis, n (%)	17 (14.8%)	16 (19.3%)	0.402
AST (U/L)	32.9 ± 46.9	28.14 ± 19.5	0.412
ALT (U/L)	39.8 ± 83.0	31.6 ± 32.3	0.390
Total bilirubin (mg/dL)	0.63 ± 0.39	0.71 ± 0.46	0.190
Platelet × 10^3^/μL	250.2 ± 81.2	250.3 ± 102.7	0.996
FIB-4	1.33 ± 0.73	1.42 ± 0.91	0.436
HBV DNA (log_10_ IU/mL)	2.84 ± 1.60	2.80 ± 1.54	0.861
Treatment duration (weeks)	58.2 ± 18.3	55.7 ± 18.6	0.340
Consolidation duration (weeks)	31.7 ± 10.7	31.1 ± 10.8	0.675

*ALT* Alanine transaminase, *AST* Aspartate transaminase, *SD* standard deviation, *TAF* tenofovir alafenamide

In the Group I, propensity score matching yielded 37 and 30 matched patients in the entecavir and TAF groups, respectively. No significant difference in clinical features was detected between the groups. The patients who discontinued TAF therapy exhibited a significantly higher rates of virological relapse (*P* = 0.014) and clinical relapse (*P* = 0.037) than did those who discontinued entecavir therapy.

### The role of HBsAg level at the time of stopping entecavir or TAF therapy in HBV relapse

In this study, HBsAg data at the time of stopping entecavir or TAF therapy (end of treatment (EOT)) was available in 133 of 144 patients who received entecavir therapy and 74 of 83 patients who received TAF therapy. Of these 207 patients, there was no significant difference in HBsAg levels at EOT between entecavir or TAF groups (2.02 ± 1.16 versus 1.84 ± 1.10 log_10_ IU/mL, *P* = 0.265). HBsAg level at EOT was an independent factor for virological relapse (HR: 1.915, 95% CI 1.749–2.480, *P* < 0.001) and clinical relapse (HR: 2.218, 95% CI 1.182–4.163, *P* = 0.013) after adjusting for other factors. Furthermore, HBsAg level at EOT was an independent factor for virological relapse in entecavir or TDF subgroups, but not clinical relapse. In the high-viremia group, HBsAg at EOT was not a significant factor for virological and clinical relapse.

### Hepatic decompensation upon clinical relapse

In accordance with the 2015 Asian Pacific Association for the Study of the Liver guidelines, we defined hepatic decompensation as a total bilirubin level of > 3.5 mg/dL (> 2.5 times the ULN [1.4 mg/dL]) and an increase in prothrombin time of > 3 s, or an INR of > 1.5 [7]. A patient in the entecavir group and two patients in the TAF group experienced hepatic decompensation upon clinical relapse. Three patients had lymphoma and underwent rituximab therapy (two patients had HBV DNA < 2000 IU/mL and one patient had HBV DNA > 2000 IU/mL at baseline). Of the three patients, one developed acute on chronic liver failure upon clinical relapse and died even though timely retreatment.

## Discussion

For patients with CHB undergoing chemotherapy and requiring antiviral prophylaxis, the risks of HBV relapse following withdrawal from antiviral prophylactic NA therapy is still present. A study reported that the cumulative incidence of HBV relapse after entecavir therapy cessation was 22.4% over a 2-years period [22]. In our previous study, the virological and clinical relapse rates 2 years after entecavir therapy cessation were 42.8% and 14.3%, respectively [23]. In the present study, the 2-years cumulative incidences of virological and clinical relapses after prophylactic entecavir therapy were 37% and 10.4%, respectively. Our results are consistent with those of other studies, including our previous one [23, 24].

Previous studies have reported that among patients with CHB, who discontinued TDF exhibited higher rates of virological and clinical relapses than did those who discontinued entecavir [16–18]. However, the literature is vague on whether different types of NA therapies lead to different HBV relapse rates after the cessation of such therapies in patients with cancer who received anti-viral prophylaxis for chemotherapy. Our previous study, which examined patients with cancer who received anti-viral prophylaxis for chemotherapy, did not reveal any significant difference in HBV relapse rates between patients who discontinued entecavir therapy and those who discontinued TDF therapy [24]. The present study indicates that the 2-year cumulative incidences of virological and clinical relapse after prophylactic TAF therapy were 46.7% and 19.5%, respectively. Compared with the patients who discontinued entecavir therapy, all those who discontinued TAF therapy exhibited a significantly higher rate of clinical relapse, and propensity score matched patients exhibited significantly higher virological and clinical relapse rates. The multivariate analysis revealed that TAF therapy was an independent factor for virological and clinical relapse after adjustment for other factors. Therefore, among the patients in the present study, those who discontinued TAF therapy exhibited higher rates of HBV relapse than did those who discontinued entecavir. The real mechanisms underlying the different relapse patterns between entecavir and TAF discontinuation remain unclear. Perhaps, TAF had a lower dose of tenofovir than TDF. TAF might have lower concentration of tenofovir in hepatocyte and HBV replication might recover rapidly after TAF withdrawal. This significant difference between the entecavir and TAF groups for the rate of HBV relapse after the cessation of prophylactic NA therapy must be further validated through prospective randomized studies.

Several risk factors are associated with HBV relapse after prophylactic NA therapy withdrawal. Studies have reported that a high baseline HBV viral load (> 2000 IU/mL), a high HBsAg level at the end of treatment, liver cirrhosis, old age (≥ 50 years), and distant metastasis were significant risk factors for HBV relapse after the cessation of prophylactic NA therapy for patients with cancer who underwent chemotherapy [23–25]. Among these risk factors, baseline HBV DNA level was the most crucial predictor of HBV relapse after the cessation of prophylactic NA therapy. The present study also observed baseline HBV DNA level to be an independent risk factor for HBV relapse after prophylactic entecavir or TAF cessation. For this reason, we divided the patients into subgroups based on their HBV DNA levels at baseline to compare their HBV relapse rates after entecavir or TAF withdrawal. The multivariate analysis revealed that TAF therapy was an independent factor for virological and clinical relapse in the high-viremia group and for clinical relapse in the moderate-viremia group. No significant difference in virological and clinical relapse rates was detected in the low-viremia groups. Thus, a high HBV relapse rate should be a cause for concern for patients with CHB who have an HBV DNA level of > 2000 IU/mL at baseline after prophylactic NA therapy (particularly with TAF) when they undergo chemotherapy. Recent studies have demonstrated that the HBsAg levels at EOT are highly predictive of a sustained response after stopping oral antiviral agents in non-cancer CHB patients [26–28]. Our previous study showed that the baseline HBV DNA and EOT HBsAg levels could predict virological relapse after withdrawal of entecavir and TDF prophylaxis for chemotherapy [24]. In this study, the serum HBsAg at EOT was available in 207 patients. HBsAg level at EOT was an independent factor for virological and clinical relapse after adjusting for other factors for all 207 patients. However, in the high-viremia group, HBsAg at EOT was not a significant factor for virological and clinical relapse. Our case number of high-viremia group is limited. Therefore, further studies are needed to investigate the role of HBsAg level at EOT in HBV relapse in high-viremia patients under NA prophylaxis for chemotherapy.

Rituximab, a chimeric mouse-human monoclonal antibody, has been used to treat patients with CD20-positive non-Hodgkin’s lymphoma. Several studies have reported high rates of HBV reactivation during cytotoxic therapy with rituximab in both patients with active HBsAg infection (HBsAg-positive) and those with resolved HBV infection (HBsAg negative but positive for antibody to hepatitis B core antigen) [29–31]. A recent study reported that rituximab-containing therapy increased the risks of HBV relapse and hepatic decompensation after withdrawal from prophylactic NA therapy [32]. In the present study, rituximab-containing chemotherapy was an independent risk factor for clinical relapses after the cessation of prophylactic NAs therapy. However, hematologic malignancy rather than rituximab was an independent factor for virological relapse. The major reason might be that the patient groups of hematologic malignancy and use of rituximab would be largely overlapped. Multivariate analysis showed that rituximab was an independent risk factor for virological relapse if hematologic malignancy is no included for analysis. However, of the 7 patients with hematologic malignancy without rituximab used, virological and clinical relapse occurred in 5 and 3 patients, respectively. Thus, HBV relapse rate was still high in patients with hematologic malignancy without rituximab used.

With respect to preventing HBV relapses, the current guidelines recommend at least 12 additional months of antiviral prophylaxis after the cessation of an anti-CD20 antibody-containing regimen [5–7, 33]. A study reported that patients who underwent anti-CD20 antibody therapy were still at risks of clinical relapse even though their NA prophylaxis treatment duration (> 12 months) was sufficient [30]. In the present study, three patients with lymphoma who initially took rituximab experienced hepatic decompensation upon clinical relapse. Moreover, one patient developed acute on chronic liver failure upon clinical relapse and died even though timely retreatment. Thus, patients with CHB who undergo a rituximab-containing cytotoxic therapy should be monitored closely after withdrawal from prophylactic NA treatment, regardless of their HBV DNA levels during initial treatment.

Previous meta-analysis presented that FIB-4 is helpful for predicting advanced fibrosis and cirrhosis in CHB patients [34, 35]. However, the role of FIB-4 in predicting HBV relapse after cessation of NA therapy is rarely reported, especially in CHB patients who received anti-viral prophylaxis for chemotherapy. In this study, FIB-4 was an independent factor for clinical relapse. In our study, all patients were non-cirrhosis. Furthermore, high AST and ALT at baseline were significant factors for HBV relapse by univariate analysis. Thus, high FIB-4 levels might be correlated with high AST and ALT levels. FIB-4 index is a useful marker for predicting clinical relapse after cessation of entecavir or TAF therapy in CHB patients who received anti-viral prophylaxis for chemotherapy.

The present study has several limitations. First, it is a single-center, retrospective study. Thus, additional multi-center and prospective studies are required verify our findings. Second, the treatment assignments were not randomized, and the entecavir and TAF groups exhibited differences in their baseline characteristics. However, being in the TAF group remained an independent factor associated with an increased rate of HBV relapse after adjustment for risk factors and propensity score matching. Third, the number of cases was limited, and the off-therapy follow-up duration could be insufficient for the TAF group.

In conclusion, HBV relapse rates were higher after the cessation of TAF than after the cessation of entecavir in patients with both cancer and HBeAg-negative CHB who underwent antiviral prophylaxis while undergoing chemotherapy, and this difference was especially pronounced for the patients in the pre-treatment high-viremia group (> 2000 IU/mL) and those who followed a rituximab-containing regimen. Thus, highly stringent and frequent monitoring and follow-up should be implemented for high-risk patients with both cancer and CHB.

## Data Availability

The datasets analyzed during the current study are not publicly available because the raw data measured by the manufacturers cannot be provided, but are available from the corresponding author on reasonable request.

## Abbreviations

ALT: Alanine aminotransferase
CHB: Chronic hepatitis B
CR: Clinical relapse
ETV: Entecavir
HBV: Hepatitis B virus
HBeAg: Hepatitis B e antigen
HBsAg: Hepatitis B surface antigen
NA: Nucleoside analogues
TAF: Tenofovir alafenamide fumarate
ULN: Upper limit of normal
